# A binder-free sulfur/reduced graphene oxide aerogel as high performance electrode materials for lithium sulfur batteries

**DOI:** 10.1038/srep39615

**Published:** 2016-12-23

**Authors:** Florian Nitze, Marco Agostini, Filippa Lundin, Anders E. C. Palmqvist, Aleksandar Matic

**Affiliations:** 1 Department of Physics, Division of Condensed Matter Physics, Chalmers University of Technology, SE-412 96 Göteborg, Sweden; 2 Department of Chemistry and Chemical Engineering, Division of Applied Chemistry, Chalmers University of Technology, SE-412 96 Göteborg, Sweden

## Abstract

Societies’ increasing need for energy storage makes it necessary to explore new concepts beyond the traditional lithium ion battery. A promising candidate is the lithium-sulfur technology with the potential to increase the energy density of the battery by a factor of 3–5. However, so far the many problems with the lithium-sulfur system have not been solved satisfactory. Here we report on a new approach utilizing a self-standing reduced graphene oxide based aerogel directly as electrodes, i.e. without further processing and without the addition of binder or conducting agents. We can thereby disrupt the common paradigm of “no battery without binder” and can pave the way to a lithium-sulfur battery with a high practical energy density. The aerogels are synthesized via a one-pot method and consist of more than 2/3 sulfur, contained inside a porous few-layered reduced graphene oxide matrix. By combining the graphene-based aerogel cathode with an electrolyte and a lithium metal anode, we demonstrate a lithium-sulfur cell with high areal capacity (more than 3 mAh/cm^2^ after 75 cycles), excellent capacity retention over 200 cycles and good sulfur utilization. Based on this performance we estimate that the energy density of this concept-cell can significantly exceed the Department of Energy (DEO) 2020-target set for transport applications.

Modern Li-ion batteries have undoubtedly revolutionized battery-powered consumer electronics. However, for large scale energy storage systems, new concepts need to be developed. It is clear that with state-of-the-art battery technologies, electric vehicles are not competitive with combustion engine powered vehicles. Key issues to address are energy and power density of the battery^1^ to allow increased driving distances, e.g. more than 500 km/charge^2^, as well as cost and sustainable solutions in terms of materials used and production methods. Cost and sustainability are also central for large-scale energy storage systems used as load leveling for intermittent power sources^3^, such as solar or wind power systems.

One of the most promising technologies for large scale energy storage is the lithium-sulfur battery^2^. With a theoretical specific capacity of 1672 mAh g^−1^ and an energy density of 2600 Wh kg^−1^, a lithium-sulfur (Li-S) battery can theoretically store energy with up to 3–5 times higher density compared to traditional lithium-ion batteries^4^,^5^. An additional advantage is the high abundance of sulfur that has the potential to significantly reduce the cost of the battery^3^ and to improve sustainability. However, the energy density is in practice significantly lower and lithium-sulfur cells commonly suffer from low lifetime^3^,^4^.

The various shortcomings of the lithium-sulfur technology are connected to the intrinsic properties of sulfur and the electrochemical reaction mechanism^4^. A Li-S cell consists of a Li-metal anode, a separator soaked in a Li^+^-conducting electrolyte, and a sulfur cathode. During discharge sulfur is converted to Li_2_S in a series of steps involving the formation of lithium polysulfides. However, the low electronic conductivity of elemental sulfur requires the addition of a conducting agent to the cathode, such as carbon, while the use of a binder, typically a polymer, is required to form a carbon sulfur composite cathode. The presence of non-active materials (carbon and polymer) typically accounts for 40–60 wt% of the cathode and reduces the practical energy density of the cell^6^,^7^,^8^. Furthermore, the low cycle life is mainly related to a large volume expansion occurring when sulfur is converted to Li_2_S and to the dissolution of lithium polysulfides, which are formed during charge and discharge, into the electrolyte. This leads to mechanical stress in the cathode, loss of active material, and shuttle reactions during cycling.

In the last decade, it has been attempted to address these three major problems by designing suitable nano-structured sulfur-carbon composite cathodes with the aim to physically contain sulfur inside a conducting matrix^6^,^7^,^9^,^10^,^11^,^12^. However, the results reported so far show that high sulfur utilization and good cycle life are difficult to combine with high sulfur loading in these composite cathodes and in addition, the practical energy density is further reduced by the use of binders.

Graphene based aerogels have many desirable characteristics for the use as electrodes in energy applications in general^13^,^14^,^15^,^16^,^17^,^18^,^19^. For Li-S batteries, properties such as light weight, high porosity and surface area are of importance. In addition aerogels can be flexible^20^ and are self-supporting structures. The self-supporting structure enables the preparation of binder-free cathodes, thereby directly increasing the practical energy density. Most concepts utilizing aerogels for Li-S battery electrodes, reported so far, have significant drawbacks such as complicated synthesis procedures and low performance^21^,^22^,^23^,^24^.

In this work, we demonstrate the direct use of a sulfur-containing graphene aerogel as a binder-free cathode in a high-energy Li-S cell. The sulfur-containing graphene-aerogel is prepared via a new, environmentally benign and straightforward synthesis route starting from graphene-oxide (GOx) and polysulfide solution as schematically shown in Fig. 1. This synthesis route combines gelling, reduction and sulfur loading in a one-pot procedure resulting in a material with very high sulfur loading, which is essential for a high energy density sulfur-based battery.

## Results

### Synthesis of the aerogel

**Figure 1:**
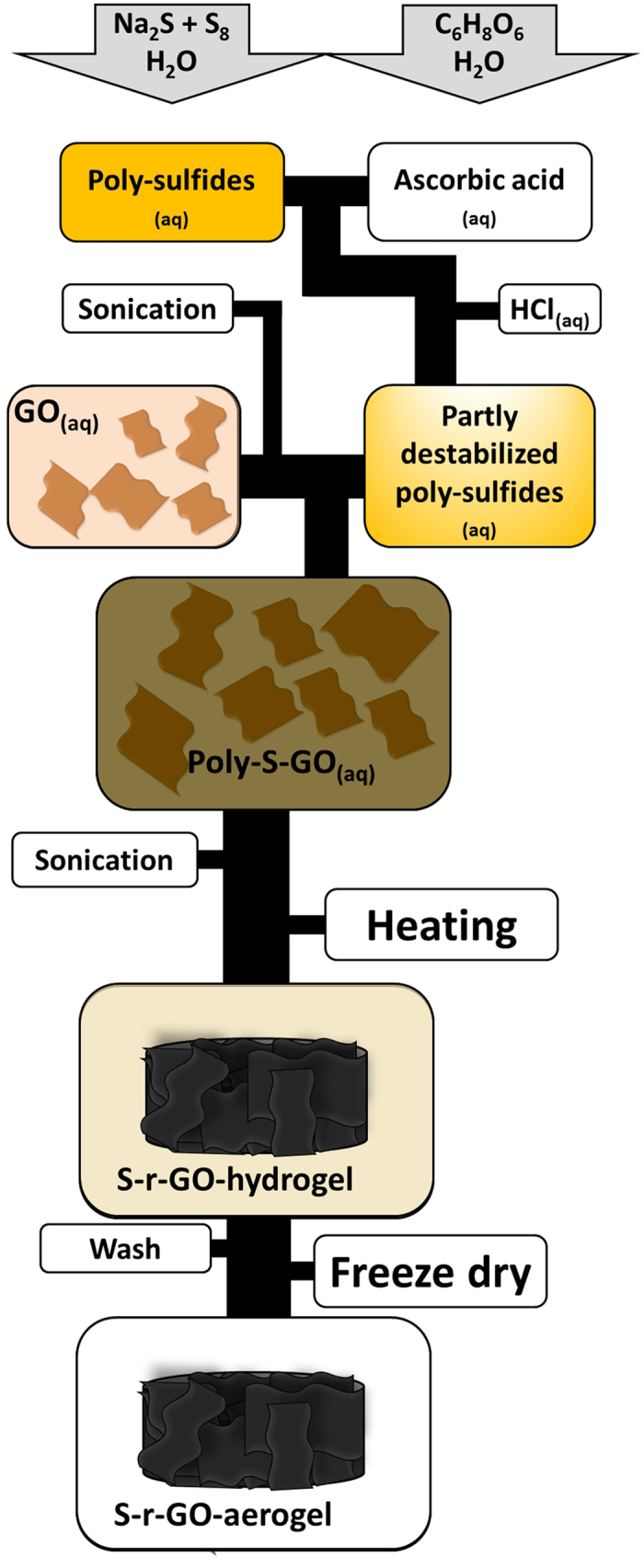
Schematic synthesis procedure of the self-standing reduced graphene oxide based high sulfur loading aerogel monoliths. The synthesis combines gelling and reduction of a polysulfide containing graphene oxide dispersion into a reduced graphene oxide aerogel. The in-situ loading with sulfur allows for preparation of binder-free cathodes with very high performance.

**Figure 2:**
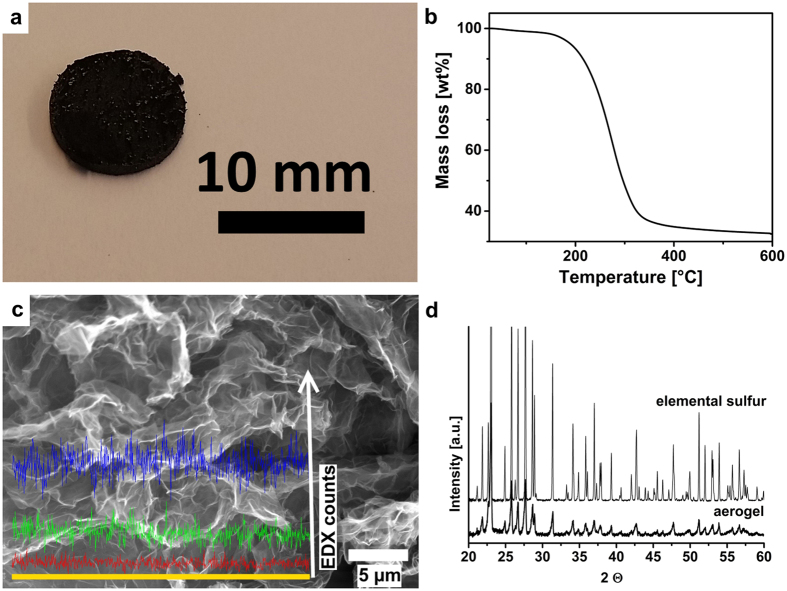
Macroscopic appearance of the aerogel and state of sulfur in the structure. (**a**) Photograph of an aerogel electrode (**b**) Relative weight loss over temperature calculated from thermogravimetric analysis data. (**c**) SEM image with EDX line scan (yellow line) over cross section surface revealing the homogenous distribution of sulfur throughout the structure. (Stronger signal in direction of the white arrow indicates stronger presence of respective element. blue: sulfur, green: carbon, red: oxygen). (**d**) X-ray diffraction pattern for sulfur-aerogel and elemental sulfur reference.

The preparation of the aerogel cathode starts from a graphene oxide dispersion and the synthesis procedure is schematically shown in Fig. 1. An aqueous polysulfide solution is destabilized by ascorbic and hydrochloric acid, resulting in finely dispersed sulfur. This sulfur containing dispersion is mixed with the graphene oxide dispersion and the mixture is sonicated and slowly heated to reduce the graphene oxide sheets. This increases the interaction between the partly reduced graphene oxide sheets as well as the interaction with the hydrophobic sulfur, leading to gelation of the dispersion, i.e. the creation of a hydrogel. The aqueous phase is then removed by freeze-drying, resulting in the final freestanding reduced graphene-oxide (r-GOx) based aerogel. The aerogel has a high sulfur loading but is nevertheless conducting thanks to the percolating graphene structure. The aerogel rods have a diameter of about 1 cm as shown in Fig. 2a. The material is in contrast to most aerogels^20^ not brittle and can be easily cut, with a regular razor blade, into discs of suitable size to be directly used as electrodes.

### Physical properties of the material

A key feature of our aerogel electrodes is the high sulfur content. Thermo-gravimetric analysis (TGA) shows a weight loss of approximately 67 wt% in the temperature range of 120–500 °C (Fig. 2b) which we relate to the loss of sulfur from the aerogel. The analysis is complicated by the fact that GOx can very well be thermally reduced in a similar temperature range^25^ and from EDX we know that there is still oxygen present. However, from the synthesis we know that the ratio of sulfur (without S from Na_2_S) to pure carbon is 5 to 1 by weight and that sulfur partly remains in the synthesis liquid. It is therefore difficult to relate all weight loss to the evaporation of sulfur. However, assuming that the oxygen solely originates from partly reduced GOx, we could estimate from dispersive X-ray spectroscopy (EDX) (see Fig. 2c and supporting information Fig. 1) the ratio of sulfur to oxygen to about 4 to 1 by weight. However, due to the vacuum conditions and the resulting error we decided against correcting for this. All calculations are therefore based on 67 wt% sulfur loading inside the aerogel. It should be mentioned that if anything, this leads to an underestimation of the performance of the final cell.

The presence of sulfur is also revealed by X-ray diffraction (XRD). The XRD pattern in Fig. 2d matches the pattern of elemental sulfur perfectly, however, the peaks are clearly broadened indicating the presence of nano-crystalline sulfur. Using Scherrer’s formula^26^ we estimate the sulfur crystallite size in the aerogel to be at least 65 nm. This size is in agreement with values in the literature for the synthesis of sulfur nanoparticles using polysulfides and acids^27^,^28^, however we have no actual information about the shape of the particles. SEM image and electron dispersive X-ray spectroscopy (EDX) scans in Fig. 2c further support the interpretation that sulfur is not incorporated as large aggregates of particles but is homogenously distributed in the structure.

**Figure 3:**
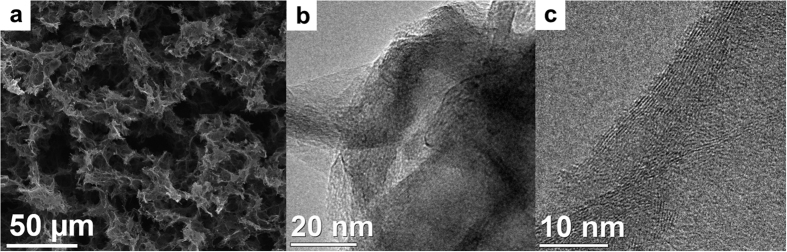
Morphology of the aerogels. (**a**) Scanning electron microscopy images of the aerogel. Transmission electron microscopy images of the aerogels showing pore walls (**b**) and wall structure (**c**) with 10–20 graphene sheets thickness.

The overall structure of the sulfur containing r-GOx aerogel, produced by this easy one-pot synthesis, is very similar to regular reduced graphene oxide based aerogels^15^,^29^. The SEM image in Fig. 3a reveals, as expected, a very porous morphology of the aerogels. However, the surface area measured by nitrogen sorption is only 33 m^2^/g. This value is low compared to sulfur-free aerogels^30^ and shows that the pores of the aerogel indeed contain a high amount of sulfur. The pore walls in the structure of the aerogel consist of 10–20 layers of reduced graphene oxide sheets as shown by high-resolution transmission electron microscopy, Fig. 3b and c.

### Use in lithium-sulfur batteries

**Figure 4:**
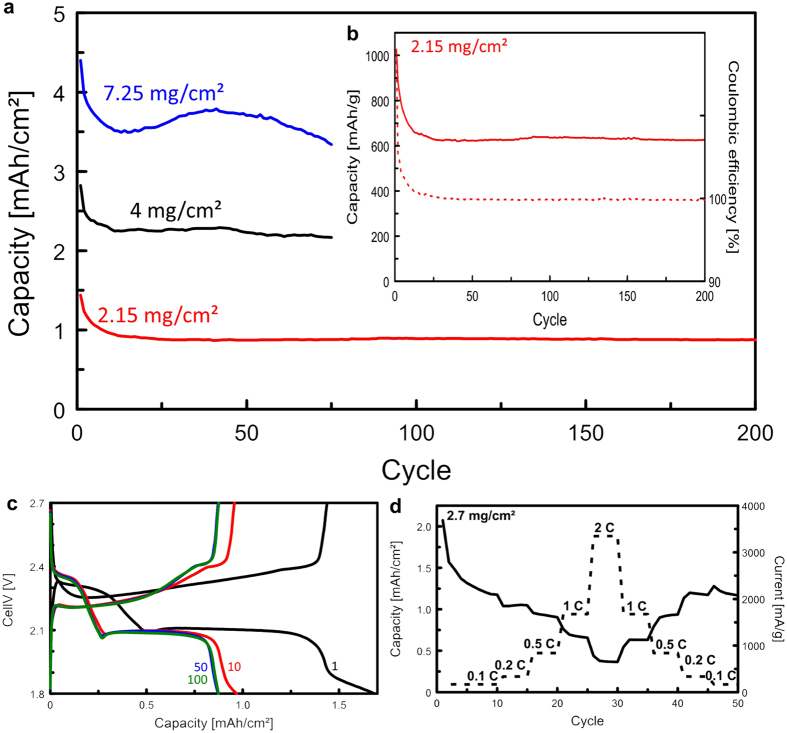
Performance of the sulfur aerogels in lithium sulfur batteries. (**a**) Total areal discharge capacity vs. cycle number at 0.1 C for different electrode mass-loading. (**b**) Inset: Specific discharge capacity (solid line) and coulombic efficiency (dashed line) vs. cycle number at 0.1 C. (**c**) Galvanostatic voltage profiles at 0.1 C. (**d**) Rate capability test performed between 0.1–2 C.

To prepare electrodes for lithium-sulfur cells the r-GOx aerogel monolith is directly cut to suitable thickness. Thus, this electrode is freestanding, binder-free and can be used directly as cathode without further processing. The electrochemical performance and cycling behavior of lithium-sulfur cells with our r-GOx aerogel cathodes are shown in Fig. 4. The overall behavior of the system is in accordance with standard lithium-sulfur electrochemistry. At discharge, a first plateau appears around 2.3 V, connected to the conversion of elemental sulfur to Li_2_S_8_ and a second plateau at 2.1 V, connected to the subsequent conversion to shorter polysulfides Li_2_S_n_ (n = 6–1). The very long plateau indicates a very high degree of direct conversion of longer polysulfides into Li_2_S thereby increasing the energy density of the cell^4^.

The initial discharge capacity (per mass sulfur in the electrode) is high, 1000–1100 mAh/g (sulfur), indicating a high degree of sulfur utilization, see in inset Fig. 4b and supporting information Fig. 2. The cycling performance in Fig. 4a shows that the capacity fades only during the first few cycles and is then remarkably stable over more than 200 cycles with excellent coulombic efficiency (99.7%) as shown in Fig. 4b. Figure 4d shows the performance of the aerogel cathodes at increased discharge rates. It reveals a good rate capability and reversibility. Increasing the rate by 20 times, from 0.1 to 2 C, results in a capacity reduction but at 1 C still about 75% of the capacity at 0.1 C can be maintained. Furthermore, the capacity is fully restored when the rate is subsequently decreased to 0.1 C, underlining the high rate capability of the system.

A particular feature of the r-GOx aerogel electrodes is that a high areal capacity can be obtained. In Fig. 4a we show that stable cycling over more than 75 cycles can be obtained for a cell with an areal capacity of more than 3.5 mAh/cm^2^ at 0.1 C. Thus, even though this is a thicker electrode the kinetics are still fast, which is a result of the high porosity and connectivity of the structure leading to high availability of the active material. However, one should note that with increased thickness of the electrodes they also become more sensitive to mechanical stress and sudden death is commonly observed between 75 and 200 cycles.

## Discussion

The high capacity and the good rate capability show the potential of the proposed r-GOx aerogel as a cathode material for lithium-sulfur batteries. For real applications, the total weight of electrodes and electrolyte is of utmost importance. Our results show that an areal capacity of more than 3.5 mAh/cm^2^ can easily be achieved. Based on this performance we estimate that the presented system can achieve a capacity per total mass (m_electrodes_  + m_electrolyte_ + m_separator_) of at least 200–300 mAh/g. This calculation is based on the ideal amount of electrolyte needed to fill the pores of the separator and the aerogel, the weight of the separator and the amount of lithium needed to balance the cell. For a lower limit scaling factor of 0.33 we considered per cm^2^: 2 mg of aerogel (50 μm thickness), 0.35 mg lithium, 0.9 mg separator (25 μm thickness), 2.75 mg electrolyte to contact all components and to fill the pores in the aerogel and the separator. The amount of electrolyte estimates that the pores of the aerogel (cumulative pore volume from nitrogen sorption: 0.21 cm^3^/g) and the separator (37 vol% of the membrane) must be filled with electrolyte multiplied with a factor of 2 to adjust for the amount of electrolyte needed to contact the components. With this assumption the here reported performance corresponds with a nominal operating voltage of 2 V to an energy density of 400–600 Wh/kg for a full cell, which is considerably higher than the Department of Energy (DEO) target^31^ of 250 Wh/kg for transport applications. We believe that the system can even be used as flexible and current collector free electrodes. However, additional studies such as in-situ XRD and test of flexible pouch cells are necessary to fully understand the behavior of the aerogels.

## Methods

### Chemicals

All chemicals were purchased from Sigma Aldrich if not specifically marked differently.

### Synthesis of aerogels

Aerogels were synthesized by the reduction and self-assembly of graphene oxide (GOx) dispersions in the presence of sodium polysulfides. A sodium polysulfide (Na-poly-S) solution was prepared from sulfur (6 g, Puriss, precipitated, 99.5–100.5%) and sodium disulfide (nonahydrate, 18 g, ACS reagent 98%) in water (240 g, distilled) by sonication and stirring overnight and mild heating (40 °C). The GOx dispersion (4 mg/ml, Graphenea, 95% monolayer) was sonicated for at least 30 min to redisperse possible deposits and to separate graphene oxide sheets into monolayers. L-ascorbic acid (80 mg, 99%) was dissolved in water (26 g, distilled) in a 40 ml glass vial with PET cap. Subsequently 4 g of Na-poly-S solution was added and hand shaken. Hydrochloric acid (0.1 ml, Puranal 37%, diluted to 5 M) was added and the vial was hand shaken. GO dispersion (10 g) was added. The vial was closed, hand shaken and sonicated at 40 °C for 30 min. The reaction was completed at 90 °C in an oven overnight for at least 8 h. The hydrogel was washed 3 times by replacing the liquid products with distilled water and allowing soluble components to diffuse out of the gel for at least 1 h. The washed hydrogel was freeze dried to remove the liquid component. The resulting aerogel was cut into discs with a razor blade. Prior to use as cathode the discs were dried in a vacuum oven at 90 °C for 1 h and directly transferred to an argon filled glove box.

### Characterization

The morphology and the chemical composition of the aerogels were characterized using an Ultra 55 FEG SEM at 10 keV equipped with an Oxford Inca EDX system. A FEI Tecnai at 200 keV was used for TEM characterization. As-synthesized aerogels were grinded and deposited dry on holey carbon film coated copper grids.

Thermogravimetric analysis was performed on a Mettler Toledo TGA/DSC 3^+^ from 25–600 °C in an alumina crucible in nitrogen flow of 20 ml/min at a heating rate of 10 °C min^−1^.

N_2_ adsorption and desorption isotherms were recorded at −196 °C using a Micromeritics Tristar. Prior to measurement each material was dried at 90 °C in vacuum for 1 h or at 45 °C in nitrogen flow for 12 h to remove adsorbed water but avoid loss of sulfur. The drying did not change the sulfur content significantly as tested by TGA. The specific surface area was calculated with the BET method.

### Electrolyte preparation

The electrolyte was prepared in an argon-filled glovebox. 1 M Lithium-bis-(trifluoromethylsulfonyl) imide (LiTFSI) (99.95%) and 0.4 M lithium nitrate (LiNO_3_) were dissolved in dimethoxyethane (DME, 99.5%) and dioxolane (DIOX, 99.8%) 1:1 vol.

### Electrochemical analysis and battery testing

Coin cells were assembled in an argon-filled glovebox using CR2032 housings and Celgard 2320 as separator. Cathodes had a mass between 1.7–5.8 mg (depending on the thickness) and a diameter of 10 mm resulting in an area of approximately 0.785 cm^2^. Lithium metal discs of 13 mm diameter were used as counter electrode. The cathodes were backed with stainless steel mesh (Goodfellow, 0.066 mm wire diameter, 37% open area). 40 μl (60 μl for the 5.8 mg electrode) of electrolyte was used per coin cell.

Coin cells were tested on a Scribner 580 battery cycler. Rates were calculated per mass sulfur in the cathode and the loading was 67 wt% sulfur per electrode. Rate capability was tested after precyling at 0.1 C for 5 cycles. Each C-rate, 0.1, 0.25, 0.5, 1 and 2 C were tested for 5 cycles followed by returning to 0.1 C. 1 C is defined as discharge of the theoretical capacity calculated from 67 wt% sulfur loading in 1 h. Stability tests were run at 0.1 C. Capacities are either specified as mAh/g with respect to mass sulfur or as mAh/cm^2^ with respect to geometric area of the electrode.

## Additional Information

**How to cite this article**: Nitze, F. *et al*. A binder-free sulfur/reduced graphene oxide aerogel as high performance electrode materials for lithium sulfur batteries. *Sci. Rep.* **6**, 39615; doi: 10.1038/srep39615 (2016).

**Publisher's note:** Springer Nature remains neutral with regard to jurisdictional claims in published maps and institutional affiliations.

## Supplementary Material

Supporting information
