# Supplementary material for: A binder-free sulfur/reduced graphene oxide aerogel as high performance electrode materials for lithium sulfur batteries
Source: Sci Rep. 2016 Dec 23;6:39615. doi: 10.1038/srep39615 (PMC5180228; doi:10.1038/srep39615)
Supplement: Supporting information [file srep39615-s1.pdf]

Supporting information

A binder-free sulfur/reduced graphene oxide aerogel as high performance electrode materials for lithium sulfur batteries

---

Florian Nitze[1,2,#], Marco Agostini[1], Filippa Lundin[1], Anders E.C. Palmqvist[2] and Aleksandar Matic[1,##]

1. Department of Physics, Division of Condensed Matter Physics, Chalmers University of Technology, SE-412 96 Göteborg, Sweden
2. Department of Chemistry and Chemical Engineering, Division of Applied Chemistry, Chalmers University of Technology, SE-412 96 Göteborg, Sweden

Corresponding author: #: florian.nitze@chalmers.se ##: matic@chalmers.se

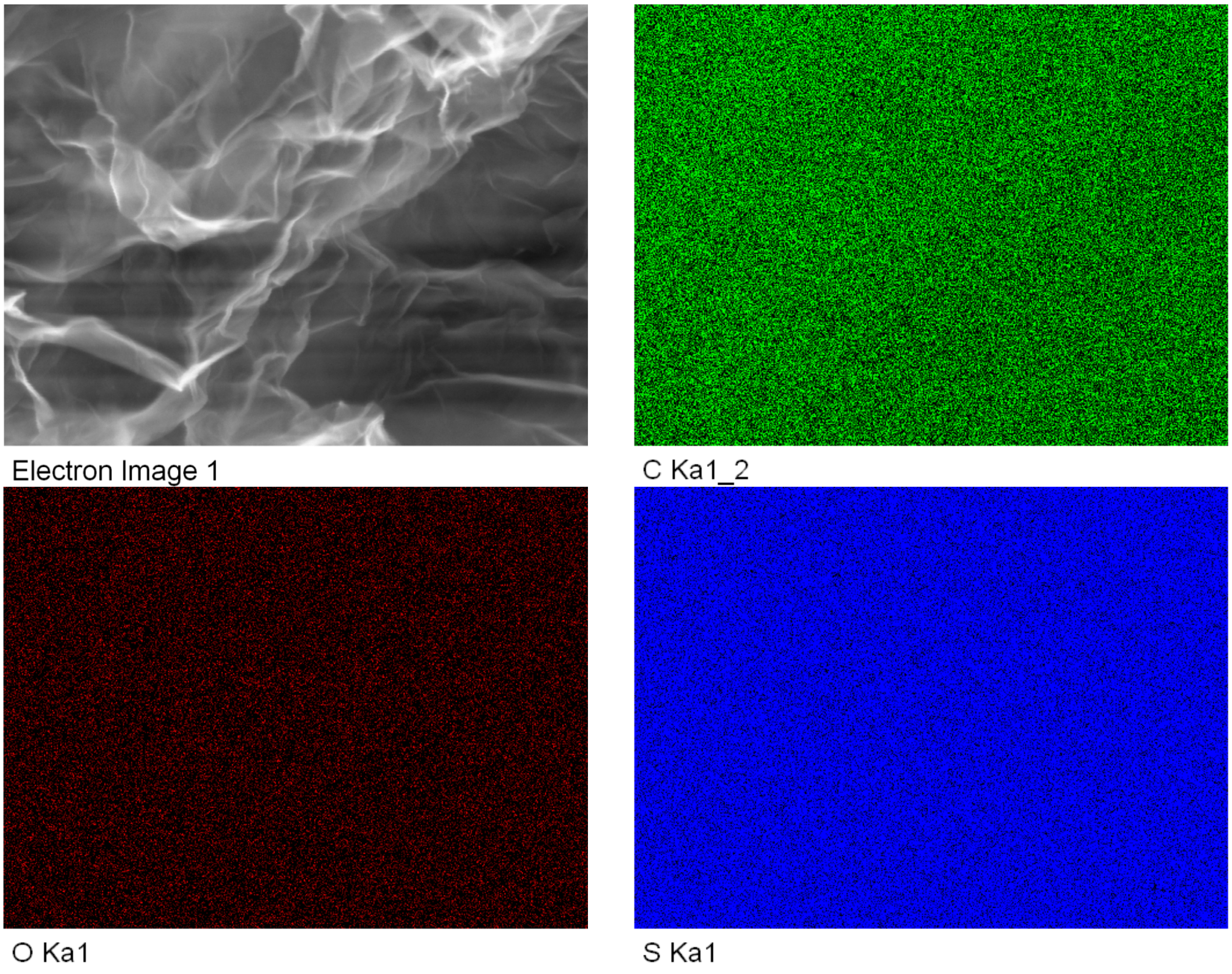

Figure 1: SEM image and EDX mapping of the aerogel.

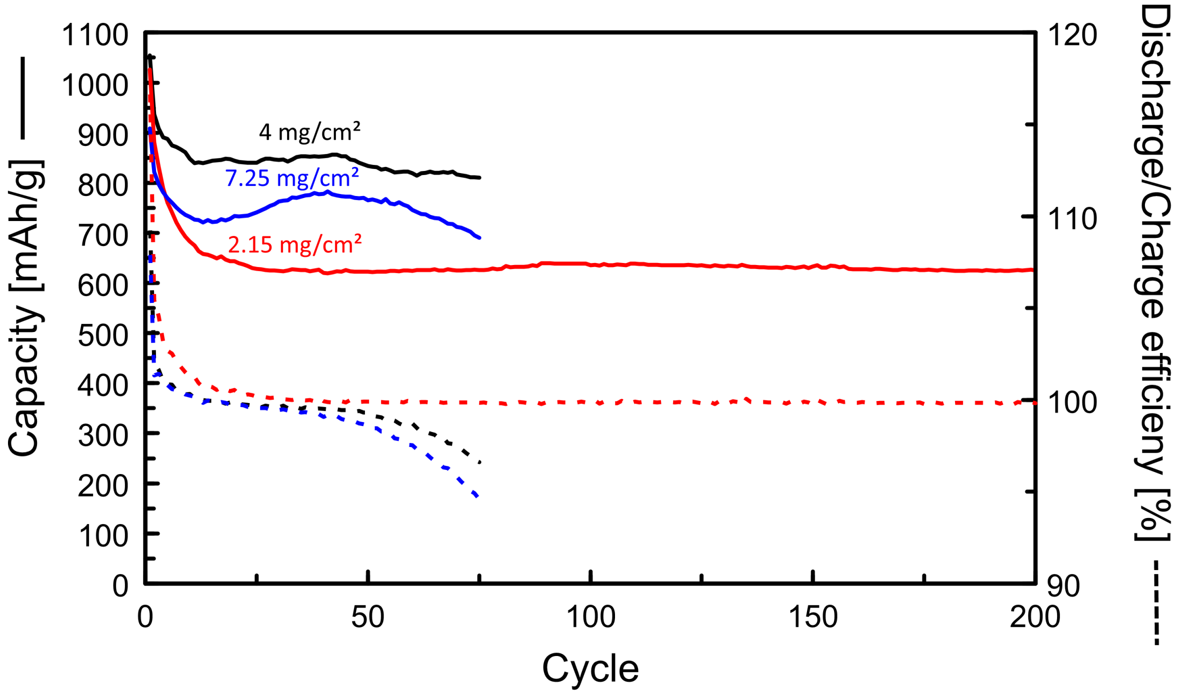

Figure 2: Specific capacity (solid line) and charge/discharge ratio (dashed line) vs. cycle number.
